# The Prevalence and Distribution of Vitreoretinal Interface Abnormalities among Urban Community Population in China

**DOI:** 10.1155/2015/742686

**Published:** 2015-11-26

**Authors:** Lei Liu, Song Yue, Jingyang Wu, Jiahua Zhang, Jie Lian, Desheng Huang, Weiping Teng, Lei Chen

**Affiliations:** ^1^Department of Ophthalmology, The First Affiliated Hospital of China Medical University, Shenyang 110001, China; ^2^Department of Healthcenter, Fengyutan Community, Shenyang 110064, China; ^3^Department of Epidemiology, School of Public Health, China Medical University, Shenyang 110122, China; ^4^Key Laboratory of Endocrine Diseases in Liaoning Province, The First Hospital of China Medical University, Shenyang 110001, China

## Abstract

The aim of this research was to identify the prevalence and distribution of vitreoretinal interface abnormalities (VIAs) among urban community population in Shenyang, China. According to the WHO criteria, a cross-sectional study was carried out among 304 Type 2 diabetes (T2D) patients and 304 people without diabetes as control over 45 years old. The presence of VIAs was determined by standardized grading of macular optical coherence tomography (Optovue OCT; Optovue, Inc., Fremont, CA) scans and two-field fundus photographs in at least one eye. For both men and women, high prevalence of VIAs (70.79%) was observed among over 65-years-old T2D patients. Prevalence of VIAs was observed to be high among T2D patients in all age groups compared to normal subjects. Prevalence of VIAs increased with age in all subjects. Prevalence of components of VIAs was epiretinal membrane (ERM) 11.43%, posterior vitreous detachment (PVD) 17.76%, vitreomacular traction syndrome (VMT) 5.67%, macular cysts/macular edema (MC/ME) 4.61%, full-thickness macular hole (FTMH) 0.82%, and partial thickness macular hole (PTMH) 0.74% in any eye, respectively. ERM and MC/ME were more prevalent in T2D in both males and females. The results highlight the need for early detection using OCT and approaches for the prevention of VIAs of diabetes in urban community.

## 1. Introduction

With the rapid development of economy, changes in lifestyle, and increasing intensification of old aging, the prevalence of diabetes was significantly rising, and the health impacts on human life became more and more serious in China [1]. According to the previous report, there were about 98.4 million people who had diabetes in 2013 and this number is predicted to be 142.7 million by 2035 in China [2]. Under this tendency, diabetic complications would be more prevalent. So we must pay more attention to prevention of various diabetic complications including diabetic eye diseases. One of the common complications of ocular in diabetes was vitreoretinal interface abnormalities (VIAs) [3]. VIAs include epiretinal membrane (ERM), vitreomacular traction (VMT), macular cysts or macular edema (MC/ME), partial thickness macular hole (PTMH), full-thickness macular hole (FTMH), and posterior vitreous detachment (PVD) [4]. PTMH and FTMH always result in visual impairment and/or blindness. In early stage, most of the VIAs were asymptomatic [5]. Therefore, early detection and screening are most important. To date, there is no research on the prevalence of VIAs in Chinese population with diabetes. This community-based, cross-sectional survey was carried out to assess the gender differences in the prevalence of VIAs among Type 2 diabetes (T2D) and normal subjects in a Chinese urban community.

## 2. Methods

### 2.1. Study Population

Fengyutan health care center is a prevention model within Liaoning Diabetic Eye Center. It provides health service for more than 80,000 residents living in five communities (including Yutan, Yonghuan, Taoyuan, Qingnian, and Zhongxin community) in Fengyutan Subdistrict, Shenyang, China. According to WHO criteria, T2D was diagnosed by general doctors and recorded in health files. Some details about this study had been reported previously [6]. Totally, 304 T2D residents and 304 normal subjects (control group) aged over 45 years who lived in Fengyutan community more than one year were selected from urban and suburb districts according to randomized resident health files. All of the selected subjects attended this study. The control group was matched for age and gender with diabetes.

### 2.2. Data Collection

Questionnaire including name, duration of diabetes, or hypertension was used to collect data. Peripheral venous blood sample was extracted over 8 h fasting. Laboratory examination including fasting plasma glucose (FPG), glycosylated hemoglobin (HbA_1_c), triglyceride (TG), and total cholesterol (TC) concentration was tested in Fengyutan health care center. Participants were seated in a darkened room. Macular scans were photographed using optical coherence tomography (Optovue OCT; Optovue, Inc., Fremont, CA). Two 45-degree nonmydriatic digital camera (Type CR6-45NM, Canon Inc., Japan) photographic fields, centered at the optic disc and macular fovea, were taken from both eyes. All images including OCT and fundus were graded in a masked manner by two ophthalmologists at the Liaoning Diabetic Eye Center separately, who were well-trained to evaluate retinal photographs and OCT images according to standardized protocols and who were masked to subjects' characteristics. If the grades were inconsistent, the other ophthalmologist would give the final decision. There were 25 subjects that could not get a clear retinal or OCT image because of lens or cornea opacity. They accepted mydriasis with tropicamide 1% (Santen Pharmaceutical Co., Ltd., Shiga, Japan) before 15 minutes of dark adaptation and binocular indirect ophthalmoscope by two ophthalmologists who reviewed retinal or OCT images. VIAs were assessed according to a standardized protocol.

Definition for VIAs was shown in Table 1.

### 2.3. Ethics Committee

The Medical Ethics Committee of the First Affiliated Hospital of China Medical University approved the research protocol of this study and all subjects gave informed written consent, according to the Declaration of Helsinki.

### 2.4. Statistical Analysis

Statistical analysis was carried out using a statistical software package (SPSS version 20.0, Chicago, IL). Descriptive statistics for continuous variables were determined as the mean ± standard deviation (SD). Otherwise, ratios and percentages for categorical variables were computed. The prevalence estimates were calculated. Chi-square test was used to determine the differences of VIAs prevalence between age groups. A *P* value <0.05 was considered to indicate statistical significance.

## 3. Results

**Figure 1:**
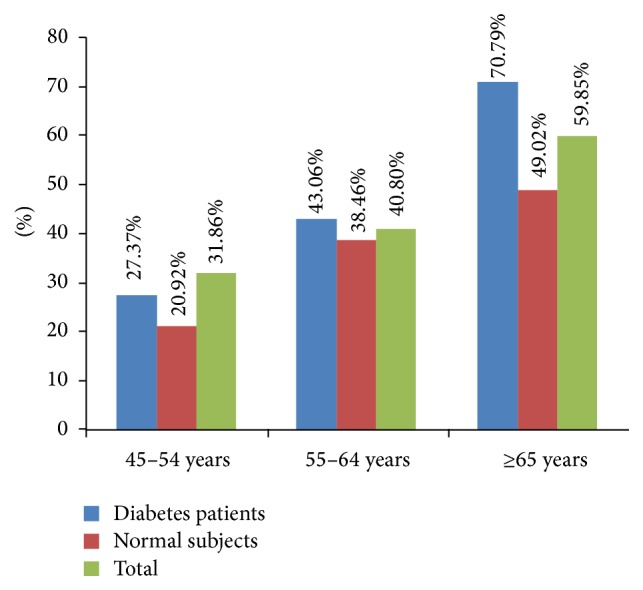
The prevalence of vitreoretinal interface abnormalities in different age groups.

**Figure 2 fig2:**
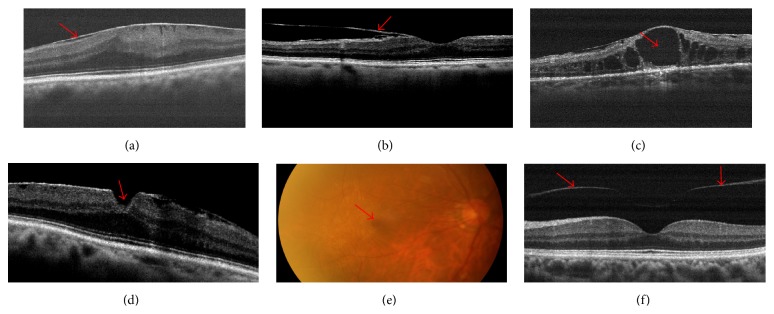
(a) Representative epiretinal membrane (ERM) image; (b) vitreomacular traction syndrome (VMT) image; (c) representative macular cysts/macular edema (MC/ME); (d) representative full-thickness macular hole (FTMH) image and representative partial thickness macular hole (PTMH) image; (e) representative posterior vitreous detachment (PVD) image. The red arrows indicate abnormities of fundus diseases.

According to Figure 1 [Fig fig2], the high prevalence of VIAs (70.79%) was observed among over 65-year-old T2D patients. Prevalence of VIAs was observed to be high among T2D patients in all age groups compared to normal subjects. The prevalence of VIAs was increased with age in all subjects. Both in males and in females, prevalence of components of VIAs was epiretinal membrane (ERM) 11.43%, posterior vitreous detachment (PVD) 17.76%, vitreomacular traction syndrome (VMT) 5.67%, macular cysts/macular edema (MC/ME) 4.61%, full-thickness macular hole (FTMH) 0.82%, and partial thickness macular hole (PTMH) 0.74% in any eye, respectively.

By comparison, no statistical difference was found in age, gender, and hypertension history between T2D patients and normal subjects (Table 2). We have found significant difference among two groups in FPG, TG, TC, and HbA_1_c levels.

In males, the prevalence of ERM (19.31%), FTMH (1.10%), VMT (9.47%), and MC/ME (9.30%) was higher in T2D patients compared with normal subjects (*P* = 0.001). There was no significant statistical difference in the prevalence of ERM and MC/ME among three age groups both in T2D patients and in normal subjects groups. However, there was a significant association between the prevalence of PVD and age increasing both in normal subjects and in T2D patients. The prevalence of VMT was increased with age in T2D patients groups (Table 3).

In females, the prevalence of VIAs including ERM (13.08%) and MC/ME (6.39%) was higher in T2D patients compared with normal subjects. The prevalence of ERM and MC/ME was increased with age in T2D patients groups. There was no significant statistical difference in the prevalence of VMT and PVD among three age groups both in T2D patients and in normal subjects groups. In addition, there was no significant statistical difference in the prevalence of PTMH, FTMH, VMT, and PVD between normal subjects and T2D patients. The results were shown in Table 4. The prevalence of VIAs within all subjects in this study was shown in Table 5.

## 4. Discussion

To the best of our knowledge, this is the first study about prevalence of VIAs in subjects within Chinese urban residents. Strength of this research is the rate of gradable quality images. There were only 3.5% of images which were unreadable, with 96.5% of participants having gradable photographs at least in one eye. There were 70.79% diabetes patients over 65 years old with VIAs. That is to say, the possibility for diabetes with older age may be much higher compared with normal subjects. Not surprisingly, increasing age was significantly associated with VIAs. This has been reported by some other studies in the past [4, 7, 8].

Previous study reported that persons with diabetes were more likely to have ERM than persons without diabetes. It was consistent with our results. In many population-based studies, diabetes was a significant risk factor for ERM [9, 10].

Snead et al. [11] report the overall prevalence of PVD to be 57% in normal subjects. In addition, the prevalence of PVD in T2D was 63.3% [12]. The previous prevalence rates of PVD were higher compared with our results. This may be because of different checking methods (we did not use B-ultrasound) and different population. The prevalence of PVD in our study subjects was higher in males than that in females both in diabetes patients and in normal subjects. However, Khalatbari et al. reported that there was no significant discrepancy in the rates of posterior ocular disease according to different sex [13].

To date, there is no related report on the discrepancy of VMT prevalence in different age groups between males and females. From Table 5, we can conclude that VMT in all DM was increased with age. It was in accordance with the prevalence of VMT in male DM. However, this tendency was not seen in female DM patients. It may be due to gender differences. But further observational studies are needed to draw a conclusion in different ethnicity or area.

In our study, we did not investigate the prevalence of macular cysts and macular edema (MC/ME), respectively. Previous study reported that the overall weighted prevalence of ME was 3.8% in US diabetes and there was no difference in the prevalence of ME by age or sex [14]. This was similar to our results.

There were some limitations in our study. As a cross-sectional study, we only evaluated the prevalence for VIAs. The causality between VIAs and diabetes could in the future be explored using a prospective study design. In addition, the risk factors for VIAs were not analyzed in this study. Thirdly, because it was carried out in community, we only used OCT and fundus camera to detect abnormalities.

In summary, in this study, we investigated the prevalence of VIAs in diabetes and in normal subjects and analyzed the discrepancy within sex. There is the need for early detection using OCT and fundus camera approaches for the prevention of VIAs in China urban community.

## Figures and Tables

**Table 1:** Definition for VIAs.

	Signs on OCT Scan	Representative
ERM	Which is characterized by hyperreflectivity of the membrane with corrugation on the surface of the internal membrane.	Figure 2(a)

Vitreomacular traction	Which was defined by the fact that the posterior hyaloid had detached from the retina but remained adhered to the retina in at least one location.	Figure 2(b)

Macular cysts	Which were defined as one or more cavities with well-defined margins within the retina that were often within low reflection.	Figure 2(c)

FTMHs	Which were considered as having hole in fovea including FTMH (steep, wide, foveal contours) and PTMH (lamellar).	Figure 2(d)

PVD	Which was defined by the fact that the posterior vitreous had detached from the retina without any adhesion.	Figure 2(e)

VIAs: vitreoretinal interface abnormalities.

**Table 2:** Characteristics for all subjects.

	All participants	Type 2 diabetes patients	Normal subjects	*P* value^*∗*^
Age, *n* (%)	608	304 (50.00)	304 (50.00)	
45–54 years	193 (31.74)	95 (31.25)	98 (32.24)	0.93
55–64 years	212 (34.87)	108 (35.52)	104 (34.21)	
≥65 years	203 (33.39)	101 (33.22)	102 (33.55)	
Sex, *n* (%)				
Females	340 (55.92)	172 (56.58)	168 (55.26)	0.74
Males	268 (44.08)	132 (43.42)	136 (44.74)	
Duration of DM, *n* (%)				
<5 years	125 (41.12)	125 (41.12)	N/A	N/A
6–15 years	101 (33.22)	101 (33.22)	N/A	
≥16 years	78 (25.66)	78 (25.66)	N/A	
Hypertension, *n* (%)				
Absent	270 (30.87)	125 (41.12)	145 (47.69)	0.10
Present	338 (69.13)	179 (58.88)	159 (52.30)	
FPG, mmol/L, mean ± SD	7.96 ± 2.43	9.23 ± 3.47	5.25 ± 1.08	<0.01
TG, mmol/L, mean ± SD	1.87 ± 0.45	1.99 ± 0.53	1.82 ± 0.34	<0.01
TC, mmol/L, mean ± SD	5.28 ± 1.68	5.79 ± 1.20	4.98 ± 1.45	<0.01
HbA_1_c, %	7.88 ± 1.05	10.11 ± 1.76	5.78 ± 0.83	<0.01

Fasting plasma glucose (FPG); glycosylated hemoglobin (HbA_1_c); triglyceride (TG); total cholesterol (TC).

^*∗*^ *P* value showed difference between Type 2 diabetes patients and normal control groups.

**Table 3:** The prevalence of VIAs within males in this study (number of eyes or %).

Variables	Type 2 diabetes patients					Normal subjects					
	45–54 years	55–64 years	≥65 years	*P* value^*∗*^	Total	45–54 years	55–64 years	≥65 years	*P* value^#^	Total	*P* value^†^
	(*n* = 74)	(*n* = 104)	(*n* = 86)		(*n* = 264)	(*n* = 98)	(*n* = 94)	(*n* = 80)		(*n* = 272)	
ERM	15 (20.3)	17 (16.3)	19 (22.09)	0.58	51 (19.31)	5 (5.10)	11 (11.70)	10 (12.5)	0.16	26 (9.56)	0.001
PTMH	0	1 (0.96)	2 (2.32)		3 (1.10)	1 (1.02)	0	1 (1.25)		2 (0.73)	0.62
FTMH	1 (1.35)	1 (0.96)	1 (1.16)		3 (1.10)	0	0	1 (1.25)		1 (0.38)	0.03
VMT	4 (5.41)	6 (5.77)	15 (17.44)	0.008	25 (9.47)	3 (3.06)	3 (3.19)	6 (7.5)	0.27	12 (4.41)	0.03
MC/ME	3 (4.05)	6 (5.77)	8 (9.30)	0.37	17 (6.44)	1 (1.02)	2 (2.12)	4 (5.00)	0.23	7 (2.57)	0.03
PVD	10 (13.51)	28 (26.92)	37 (43.02)	<0.01	75 (28.4)	13 (13.27)	37 (39.36)	40 (50.00)	<0.01	90 (33.09)	0.24
VIAs	33 (44.59)	59 (56.73)	82 (95.35)	<0.01	174 (65.9)	23 (23.47)	53 (56.38)	62 (77.5)	<0.01	138 (50.74)	<0.01

Epiretinal membrane without schisis (ERM); macular cysts/macular edema (MC/ME); partial thickness macular hole (PTMH); full-thickness macular hole (FTMH); posterior vitreous detachment (PVD); vitreomacular traction syndrome (VMT).

^*∗*^Difference analysis between three age groups in Type 2 diabetes patients.

^#^Difference analysis between three age groups in normal subjects.

^†^Difference analysis between Type 2 diabetes patients and normal subjects groups.

**Table 4:** The prevalence of VIAs within females in this study (number of eyes or %).

Variables	Type 2 diabetes patients					Normal subjects (number of eyes)					
	45–54 years	55–64 years	≥65 years	*P* value^*∗*^	Total	45–54 years	55–64 years	≥65 years	*P* value^#^	Total	*P* value^†^
	(*n* = 116)	(*n* = 112)	(*n* = 116)		(*n* = 344)	(*n* = 98)	(*n* = 114)	(*n* = 124)		(*n* = 336)	
ERM	5 (4.31)	15 (13.39)	25 (21.55)	<0.01	45 (13.08)	7 (7.14)	9 (7.89)	11 (8.87)	0.89	27 (8.03)	0.03
PTMH	1 (0.86)	1 (0.89)	1 (0.86)		3 (0.87)	0	0	1 (0.81)		1 (0.29)	0.63
FTMH	1 (0.86)	0	2 (1.72)		3 (0.87)	0	2 (1.75)	1 (0.81)		3 (0.89)	0.73
VMT	4 (3.44)	6 (5.36)	9 (7.76)	0.35	19 (5.52)	3 (3.06)	4 (3.51)	6 (4.84)	0.76	13 (3.87)	0.31
MC/ME	3 (2.58)	5 (4.46)	14 (12.07)	<0.01	22 (6.39)	0	3 (2.63)	7 (5.65)		10 (2.98)	0.03
PVD	5 (4.31)	7 (6.25)	10 (8.62)	0.40	22 (6.39)	8 (8.16)	9 (7.89)	12 (9.68)	0.87	29 (8.63)	0.26
VIAs	19 (16.38)	34 (30.35)	61 (52.59)	<0.01	114 (33.14)	18 (18.37)	27 (23.68)	38 (30.64)	<0.01	83 (24.70)	<0.01

Epiretinal membrane without schisis (ERM); macular cysts/macular edema (MC/ME); partial thickness macular hole (PTMH); full-thickness macular hole (FTMH); posterior vitreous detachment (PVD); vitreomacular traction syndrome (VMT).

^*∗*^Difference analysis between three age groups in Type 2 diabetes patients.

^#^Difference analysis between three age groups in normal subjects.

^†^Difference analysis between Type 2 diabetes patients and normal subjects groups.

**Table 5:** The prevalence of VIAs within all subjects in this study (number of eyes or %).

Variables	Type 2 diabetes patients					Normal subjects					
	45–54 years	55–64 years	≥65 years	*P* value^*∗*^	Total	45–54 years	55–64 years	≥65 years	*P* value^#^	Total	*P* value^†^
	(*n* = 190)	(*n* = 216)	(*n* = 202)		(*n* = 608)	(*n* = 196)	(*n* = 208)	(*n* = 204)		(*n* = 608)	
ERM	20 (10.52)	32 (14.81)	44 (21.78)	<0.01	96 (13.95)	12 (6.12)	20 (9.62)	21 (10.29)	0.28	53 (8.72)	<0.01
PTMH	1 (0.52)	2 (0.93)	3 (1.48)	0.62	6 (0.87)	1 (0.51)	—	2 (0.98)	—	3 (0.49)	0.31
FTMH	2 (1.05)	1 (0.46)	3 (1.48)	0.56	6 (0.87)	—	2 (0.86)	2 (0.98)	—	4 (0.66)	0.52
VMT	8 (4.21)	12 (5.56)	24 (11.88)	<0.01	44 (6.39)	6 (3.06)	7 (3.36)	12 (5.88)	0.29	25 (4.11)	0.02
MC/ME	6 (3.15)	11 (5.09)	22 (10.89)	<0.01	39 (5.66)	1 (0.51)	5 (24.04)	11 (5.39)	0.01	17 (2.79)	<0.01
PVD	15 (7.89)	35 (16.20)	47 (23.26)	<0.01	97 (14.09)	21 (10.71)	46 (22.12)	52 (25.49)	<0.01	119 (19.57)	0.09
VIAs	52 (27.37)	93 (43.06)	143 (70.79)	<0.01	288 (41.86)	41 (20.92)	80 (38.46)	100 (49.02)	<0.01	221 (36.35)	<0.01

Epiretinal membrane without schisis (ERM); macular cysts/macular edema (MC/ME); partial thickness macular hole (PTMH); full-thickness macular hole (FTMH); posterior vitreous detachment (PVD); vitreomacular traction syndrome (VMT).

^*∗*^Difference analysis between three age groups in Type 2 diabetes patients.

^#^Difference analysis between three age-groups in normal subjects.

^†^Difference analysis between Type 2 diabetes patients and normal subjects groups.
